# Assessing body composition using auto-segmentations of muscle and subcutaneous adipose tissue in prostate cancer patients receiving magnetic resonance-guided radiotherapy

**DOI:** 10.1016/j.phro.2025.100882

**Published:** 2025-12-06

**Authors:** Aaron G. Rankin, Dónal M. McSweeney, Jim Zhong, Carlos Garcia Escobar, William Russell, Angela Davey, Marianne C. Aznar, Alan McWilliam

**Affiliations:** a Division of Cancer Sciences, University of Manchester, Manchester, UK; b Department of Diagnostic and Interventional Radiology, Leeds Teaching Hospitals NHS Trust, Leeds, UK; c Leeds Institute of Medical Research, University of Leeds, Leeds, UK

**Keywords:** Prostate Cancer, MR-guided Radiotherapy, Body Composition, Auto-segmentation, Sarcopenia, Deep learning

## Abstract

**Background and purpose:**

Sarcopenia negatively impacts treatment outcomes in prostate cancer (PCa) patients. With the growing use of magnetic resonance-guided radiotherapy (MRgRT), automated body composition (BC) analysis using deep learning frameworks may allow for early detection of at-risk patients and intervention during treatment.

**Methods:**

T2-weighted images were obtained from 71 patients with intermediate-risk PCa who underwent MRgRT using one of three treatment regimens: androgen deprivation therapy (ADT) with 60 Gy in 20 fractions (20# + ADT), ADT with 36.25 Gy in 5 fractions (5# + ADT), or 36.25 Gy in 5 fractions without ADT (5# + noADT). An auto-segmentation model was developed using a 36/9 train–test split with 5-fold cross-validation, implemented with and without a bowel and bone (BB) mask, to delineate muscle and subcutaneous adipose tissue (SAT). Performance was assessed using mean surface distance, 95th percentile Hausdorff distance and Dice similarity coefficient. The model was then applied to quantify muscle, SAT, and intramuscular adipose tissue from images acquired during MRgRT.

**Results:**

Performance metrics were similar with and without the BB mask; however, using the mask resulted in a 24 % reduction in failures. At the start of MRgRT, SAT area differed significantly between 20# + ADT and 5# + noADT (p = 0.04), with significant changes across all ROIs observed by treatment end.

**Conclusion:**

Routinely acquired MRgRT images can be used for reliable auto-segmentation of BC metrics. Significant differences in BC metrics were found between treatment regimens. This supports their use for sarcopenia detection and adaptive treatment planning.

## Highlights

• First use of on-treatment magnetic resonance images to assess body composition.
• Deep-learning model reached expert-level muscle and adipose tissue segmentation.
• Significant changes were observed between prostate cancer treatment groups.
• Supports routine sarcopenia monitoring during radiotherapy.

## Graphical abstract

**Figure:**
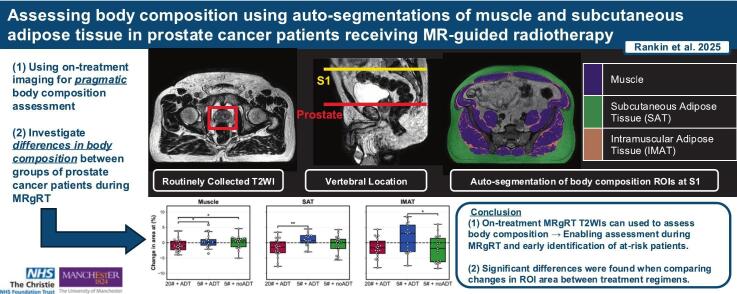

## 1 Introduction

Sarcopenia is the progressive loss of muscle mass and function, classified as primary when age-related and secondary when driven by underlying conditions such as cancer [1,2]. Studies have shown that sarcopenic patients have decreased survival and increased toxicity in numerous cancers [3]. Sarcopenia is also associated with increased risk of fractures and a loss of independence as patients become increasingly frail and vulnerable [4]. Additionally, it directly impacts the quality of life of patients following treatment, alongside predisposing patients to worse treatment outcomes [5].

Medical imaging provides clinical insight into the underlying anatomy, and segmentations of regions of interest (ROIs) can aid in the development of image-based biomarkers [6]. In recent years, deep learning (DL) models have been shown to be increasingly capable of producing segmentations of comparable standard to expert clinicians – reducing the time burden on medical professionals [7]. As a result, multiple studies have demonstrated the utility of DL models to auto-segment skeletal muscle and adipose tissue both in computed tomography (CT) and magnetic resonance (MR) images [[7], [8], [9]]. Such models also enable large scale retrospective analysis of routinely collected imaging data. With the increasing availability of magnetic resonance-guided radiotherapy (MRgRT), particularly in prostate cancer (PCa) treatment, there is an opportunity to analyse anatomically detailed images during radiotherapy (RT). Early detection of adverse changes in muscle and fat mass during MRgRT may improve treatment outcomes and post-treatment quality of life. Baseline assessment of body composition (BC) can highlight patients who may already be at risk due to low muscle mass or high fat infiltration, enabling interventions such as nutritional support or resistance training to maintain muscle mass and function throughout treatment [4,10]. Monitoring changes over the course of treatment may reveal escalating risk, either due to treatment-related physical deterioration in vulnerable patients or driven by systemic effects of the disease itself, such as cancer-associated cachexia [11].

In PCa, sarcopenia is associated with worse treatment outcomes, but there is limited research on assessing sarcopenia using magnetic resonance imaging (MRI) or during RT [12]. One study conducted by McDonald et al., showed that assessing muscle mass and bone density measured at L5 could predict non-cancer related deaths in localised PCa patients following RT [13]. Androgen deprivation therapy (ADT), a mainstay of modern PCa care, is known to reduce muscle mass by suppressing testosterone and dihydrotestosterone, hormones essential for maintaining skeletal muscle [[14], [15], [16], [17], [18]]. Understanding whether changes in BC can be detected during MRgRT for PCa is important, as this could provide valuable insights for optimising treatment and patient management.

In this work, we developed the first machine-learning model capable of segmenting muscle and subcutaneous adipose tissue (SAT) compartments from routinely acquired MR-linac images. These segmentations were used to assess in a proof-of-principle analysis evaluating firstly, baseline measures of muscle and fat mass, and secondly, if longitudinal changes in BC during treatment are evident for three patient groups undergoing distinct treatment regimens with MRgRT.

## 2 Materials and methods

### 2.1 Patient population

Images from 71 patients with localised intermediate-risk PCa were analysed. All patients were treated and imaged on the 1.5 T Elekta Unity MR-linac system and were recruited as part of an ongoing prospective observational imaging trial (ClinicalTrials.gov identifier: NCT30500081). All patients provided informed consent, and the study was approved by the institutional research ethics board in accordance with the Declaration of Helsinki. Patients were grouped by three treatment regimens: ADT and RT with a total dose of 60 Gy delivered in 20 fractions (20# + ADT, n = 28); ADT and RT with 36.25 Gy in five fractions (5# + ADT, n = 21); and five fractions of RT without ADT (5# + noADT, n = 22). For patients treated with ADT, treatment began at least three months prior to the first fraction of RT.

For patients treated with five fractions, fractions were delivered every second day over two working weeks and for patients treated with 20 fractions, MRgRT was administered daily over four working weeks. T2-weighted images were acquired at each treatment fraction for patients treated with five fractions as part of the MRgRT workflow, whereas weekly images were obtained for patients treated with 20 fractions. Full image acquisition parameters are detailed in Supplementary Table S1.

### 2.2 Segmentation model development

#### 2.2.1 Data preparation

Fifteen patients were arbitrarily selected from each of the treatment regimens and an MRI acquired during the first fraction of MRgRT was collected. A specialist radiologist with 10 years of experience manually delineated all skeletal muscle compartments and SAT using RayStation 11B (RaySearch Laboratories, Stockholm, Sweden). Segmentations were performed at the base of the sacrum (S1 level) on a single axial slice on images acquired during the first treatment as S1 was within the field of view for on-treatment imaging of PCa patients.

**Fig. 1:**
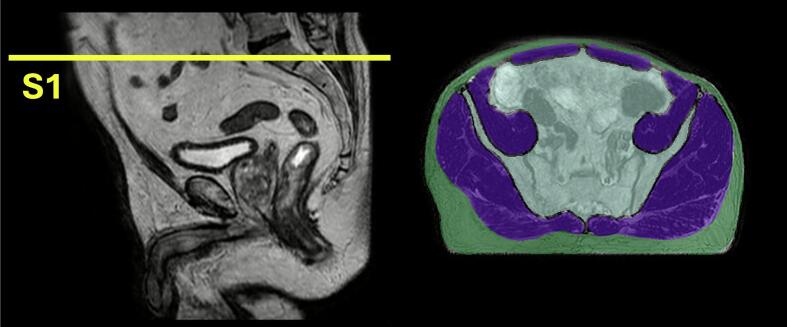
Segmentation location and ROIs (left) Sagittal view showing the location the sacrum (S1) and the location of the axial 2D slice where segmentations were performed. (right) Example of axial slice used in model training with each ROI highlighted: muscle (purple), subcutaneous adipose tissue (green) and bowel and bone (blue). Note: not shown here is intramuscular adipose tissue. This was added in post-processing during longitudinal analysis and not used during the development of the segmentation model. (For interpretation of the references to colour in this figure legend, the reader is referred to the web version of this article.)

To improve muscle segmentation, a bowel and bone (BB) mask was generated by subtracting the segmented SAT and muscle from the convex hull of the SAT mask, isolating internal structures such as bowel and bone. This reduced misclassification of bowel gas as muscle and was incorporated as an additional input channel during model training to provide anatomical context. Fig. 1 illustrates the selected 2D slice and corresponding axial view with the ROIs used in model development.

#### 2.2.2 Model training

A UNet-based segmentation model was implemented using the *Segmentation Models PyTorch* library, building on a previously developed in-house framework [[19], [20], [21], [22]]. The five-level encoder–decoder network with skip connections, produces multi-class segmentation masks. A *MixNet* backbone was used for the encoder, with weights initialised from ImageNet [23]. Inputs were single axial slices (480 × 480 pixels) at the S1 level. A further breakdown of technical details, alongside a CLAIM checklist to support reproducibility, are provided in Supplementary Material Section 2.2.

Training used the Adam optimizer with an initial learning rate of 3 × 10 − 4, and the loss function combined categorical cross-entropy with multiclass Dice [24]. Data augmentation included random rotations and horizontal flips. Model development employed 5-fold cross-validation: the dataset was split into five folds (nine images each), with three folds for training, one for validation, and one for evaluation, rotated so each patient appeared once in the evaluation set. Performance metrics were averaged for the five models [25]. To assess the impact of the BB mask, two variants were trained: Model A (with BB mask) and Model B (without).

#### 2.2.3 Model evaluation

Model performance was assessed using mean surface distance (MSD), the 95th percentile Hausdorff distance (HD95) and Dice similarity coefficient (DSC) for muscle and SAT segmentations. The trained models were subsequently applied to the full dataset of 350 scans, and all outputs underwent visual inspection. Scans with segmentation errors were excluded from further analysis. In addition to quantitative metrics, the frequency and nature of segmentation failures were documented and compared between Model A and Model B.

## 3 Body composition analysis

The best-performing segmentation model was applied to the remaining scans to evaluate longitudinal changes in adipose tissue and muscle during MRgRT. For consistency, each image was rigidly registered to the first-fraction scan *for each* patient*.* The S1 level was predefined by an expert radiologist on the first-fraction image, and segmentations were performed on three axial slices (S1 ± 1 slice).

For each scan, the mean cross-sectional area of SAT, intramuscular adipose tissue (IMAT), and muscle was calculated. IMAT was derived in post-processing from the muscle mask, by thresholding pixels with intensities greater than the mean + 1 standard deviation. This was included to refine lean muscle quantification and potentially capture treatment-related IMAT changes, which have been associated with poorer outcomes [26,27].

Relative changes in areas were expressed as percentage differences from first-fraction values. Any ROI with > 10 % relative change was deemed an outlier and removed from analysis. Differences in baseline areas and longitudinal changes across treatment regimens were tested using Mann–Whitney U tests. All analyses were performed in Python (version 3.10) with *SciPy* (version 1.13) [28].

## 4 Results

### 4.1 Patient population

A breakdown of the number of patients included in the study per treatment group and details relating to treatment characteristics can be found in Table 1. One patient from the 20# + ADT group was excluded from analysis due to poor registration in images acquired during treatment.

**Table 1:** Breakdown of patient characteristics in treatment groups and number of patients involved in the study. Values quoted are the median and range within square brackets.

Variable		20# + ADT (n = 28)	5# + ADT (n = 21)	5# + noADT (n = 22)	p-val^a^
Age (years)		70	69.5	67	0.087
		[55–75]	[63–77]	[44–76]	
Gleason Score	3 + 3	0	0	2	0.041
	3 + 4	13	14	18	
	4 + 3	8	7	2	
	4 + 4	6	0	0	
T-Stage	1	1	3	5	0.019
	2	18	16	17	
	3	9	2	0	
RT Duration (days)		27	10	10	
		[25,30]	[9,15]	[9,15]	
Patients in training set (n)		15	15	15	
Removed from analysis (n)		1	0	0	
Total number of scans used (n)		135	105	110	

a Kruskal-Wallis test used to test for differences in age, χ^2^ test used for differences in Gleason score and T-stage.

### 4.2 Segmentation performance

**Fig. 2:**
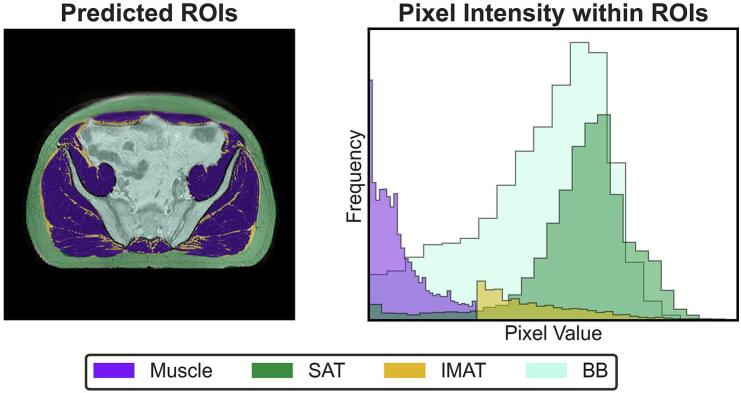
Examples of predicted ROIs and image intensities. (left) Predicted ROIs for an exemplar patient. Segmentation model outputs are muscle, subcutaneous adipose tissue (SAT) and bowel and bone (BB). Intramuscular adipose tissue (IMAT) was defined by removing pixels within the muscle mask above (mean + 1 standard deviation) to bolster lean muscle mass measurements. (right) Image intensity histogram for each of the ROIs. The BB mask was included in model training to reduce the number of nearby bowel gas pixels being classified as muscle due to their close proximity and relatively similar pixel intensities (visualised in the overlap in intensity histograms).

Model performance was assessed using MSD, HD95, and DSC. Metrics between both the model trained with the BB mask and without were largely consistent – values are displayed in Table 2. The BB mask slightly improved muscle segmentation based on MSD, while HD95 metrics showed minor decrements in performance. The small reduction in SAT segmentation accuracy likely stems from overlapping image intensities, as demonstrated by the histogram peaks in Fig. 2. DSC metrics were consistently high across models, indicating very good agreement, with muscle DSC ranging from 0.95 to 0.96 and SAT at 0.93 for both models.

**Table 2:** Segmentation metrics for muscle and subcutaneous adipose tissue (SAT) used to assess accuracy of model trained with the bowel and bone (BB) mask (Model A) and without the BB mask (Model B). The BB mask was included to improve segmentation performance for muscle by reducing the number of cases where bowel gas was incorrectly segmented as muscle. Metrics included are mean surface distance (MSD), the 95th percentile of the Hausdorff distance (HD95) and Dice Similarity Coefficient (DSC). Values quoted are the mean across 5-fold cross-validation with 95 % confidence intervals below.

ROI	Model	MSD (mm) [CI95]	HD95 (mm)[CI95]	DSC [CI95]
**Muscle**	**A**	1.56 ± 0.10 [1.37, 1.75]	4.99 ± 0.68 [3.62, 6.36]	0.96 ± 0.01 [0.94, 0.97]
	**B**	1.60 ± 0.09 [1.41, 1.79]	4.91 ± 0.64 [3.61, 6.20]	0.95 ± 0.01 [0.94, 0.96]
**SAT**	**A**	1.18 ± 0.05 [1.08, 1.27]	3.32 ± 0.17 [2.98, 3.65]	0.93 ± 0.01 [0.93, 0.94]
	**B**	1.11 ± 0.03 [1.05, 1.17]	3.04 ± 0.09 [2.86, 3.22]	0.93 ± 0.01 [0.93, 0.94]

Visual review of all 350 images showed the BB mask’s main advantage was reducing misclassification of rectal gas as muscle (see Supplementary Fig. S4). Model B failed on 43 images (12.3 %) versus 33 (9.4 %) with Model A, a 24 % reduction. However, the number of patients with at least one failure dropped only slightly (15 to 14), suggesting patient-specific factors may underlie segmentation errors. Edge artefacts were also noted in some cases. Model A was therefore selected for further analysis.

### 4.3 Body composition changes

After performing segmentations across all 350 scans, the number of cases where relative percentage changes were found to be greater than 10 % was 15, with 5 of these caught during the visual quality inspection stage. Outlier cases were most common when segmenting SAT, possibly due to edge artefacts or registration error.

At baseline, a significant difference (p = 0.04) was found when comparing SAT area between 20# + ADT and 5# + noADT patients but on comparing distributions among other ROIs, no significant differences were found. Mean areas for each ROI at the beginning and end of MRgRT are found in Supplementary Table S2.

**Fig. 3:**
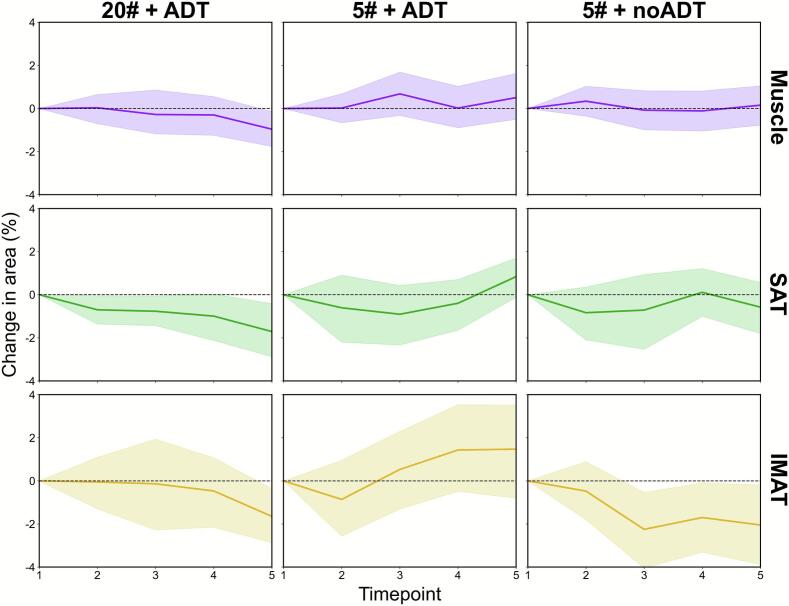
Longitudinal changes during treatment across ROIs and treatment groups. Changes in ROI area relative to fraction 1 for each treatment group. The solid line represents the mean across the treatment group and shaded regions reflect 95 % confidence intervals. Images were collected at five timepoints for each patient: weekly for patients treated with 20# (median treatment duration 27 days) and at each treatment fraction for 5# patients (median treatment duration 10 days).

Fig. 3 shows how relative ROI area changed during treatment for each treatment regime. For patients that received 20# + ADT, all ROI areas decreased by the end of treatment, with mean changes of −1.0 ± 0.4 %, −1.9 ± 0.6 % and −1.8 ± 0.7 % for muscle, SAT and IMAT respectively. The group with the smallest variation was muscle for 5# + noADT patients, where there was little change (0.2 ± 0.5 %) by the end of treatment. IMAT showed the greatest variation between treatment groups, as shown in Fig. 3. However, it is noted that patients who have not received ADT prior to RT show a rapid and consistent decrease in IMAT.

**Fig. 4:**
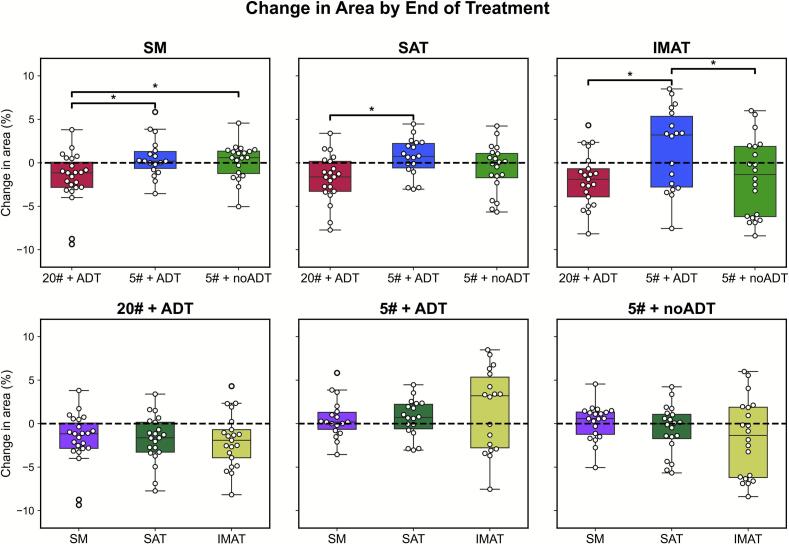
Boxplot of relative changes in area by the end of radiotherapy. Boxplots showing changes in area of ROIs between treatment regimens. Relative changes in each ROI were computed by determining the change from the first to last fraction of treatment – median treatment duration for 5# patients was 10 days and was 27 days for 20# patients. Mann-Whitney U tests were used to assess statistical significance between distributions and ** denotes a *p* ≤ 0.01, * denotes 0.01 < *p* ≤ 0.05.

Changes in area by the end of treatment are shown in Fig. 4 for each treatment group and ROI. Supplementary Fig. S6 shows changes in ROI area for 2 patients in each group across treatment. Significant differences were found in the distributions of muscle area change between both 20# + ADT vs. 5# + noADT (p = 0.04), and 20# + ADT vs. 5# + ADT (p = 0.05). Statistically significant differences were also found when comparing changes in SAT between 20# + ADT and 5# + ADT (p = 0.005). IMAT changes were also significant between 5# + ADT and 5# + noADT (p = 0.04). No significant changes were found comparing ROIs within each treatment group – where muscle decreases were not accompanied with SAT or IMAT increases.

## 5 Discussion

We demonstrated the feasibility of using automatic segmentation to assess BC in patients treated with MRgRT. To our knowledge, this is the first study to assess BC using routinely acquired MRgRT images. We developed a segmentation tool for BC assessment, demonstrating its potential for monitoring longitudinal changes during treatment. Given that sarcopenic patients experience poorer treatment outcomes and quality of life, an automated monitoring system using routine imaging could help identify at-risk individuals earlier, enabling timely interventions such as personalised nutritional support and tailored exercise programs.

In a recent review, Winder et al. found that in studies measuring BC metrics from abdominal images, CT studies outnumbered MR studies four to one [29]. While multiple studies have validated MR-based segmentation models for BC monitoring, the work presented here highlights that daily MR imaging can effectively track compartmental changes within the RT treatment window. Our model used routinely collected T2WIs from a single institution, ensuring consistent acquisition parameters – reducing the need for additional standardisation. A challenge of T2WI is the low contrast between muscle, bowel gas, and adipose tissue, complicating segmentation. Including the BB mask helped mitigate these limitations by improving muscle and bowel gas distinction during model training. Although CT-based BC studies are more prevalent, segmentation accuracy between CT and MR imaging has been shown to be comparable, supporting the use of MR as a reliable modality for longitudinal BC assessment [29,30].

Segmentation performance was evaluated using 5-fold cross-validation, comparing models with and without the BB mask. Although the mask slightly affected metrics, its primary benefit was a 24 % reduction in visual segmentation failures. The most common error, misclassification of bowel gas as muscle, was reduced with the inclusion of the mask. These errors were easily identifiable through visual inspection or flagged by analysing relative changes during treatment. Any segmentation errors could be corrected by a clinician with minor manual refinements using modern delineation tools.

For longitudinal assessments, we defined a threshold for each image to remove IMAT from muscle masks. This approach improved lean muscle mass measurements and enabled us to explore whether IMAT could be tracked using T2WI. Fat infiltration, or myosteatosis, a marker of muscle quality, has been linked to reduced quality of life and increased mortality [26,27]. Our results showed that while IMAT did not change as consistently as muscle or SAT during treatment, its variability was greater, as seen in the fluctuations in trajectory in Fig. 3. Measuring IMAT with on-treatment T2WI may hold potential for assessing the onset or progression of myosteatosis, but further studies are needed to establish a more robust analytical framework, such as incorporating fat-specific imaging protocols [10].

This study assessed BC in PCa patients using the S1 level as a proxy for total muscle mass, as the conventional L3 level was often outside the imaging field. The novelty of our approach is the use of on-treatment MR images, where S1 was consistently visible, enabling daily assessment during MRgRT. Both L3 and S1 include the psoas muscles, reliable indicators of muscle mass, and Shen et al. showed that accurate fat and muscle estimates can be obtained from a single MR slice at L4–5 [31]. Although gluteal muscles are not always visible at L3, they have also been used for BC assessments [32,33], and the adjacent L5 level, anatomically similar and directly connected to S1, has likewise been employed in BC studies, though further work is needed to clarify the relationship between S1 and L3 [34]. Beer et al. showed that psoas thickness on T1- and T2-weighted images identifies sarcopenia and predicts mortality; while future work could segment individual muscle components, total muscle remains a robust overall health measure [35,36].

Segmentations were performed by a single observer, so interobserver variability could not be assessed. However, Nowak et al. demonstrated that single-slice MRI BC measurements at L3 showed low interobserver variability and excellent intra- and inter-scanner repeatability in 12 patients [37], supporting the reliability of this approach. To account for anatomical variation, segmentations were assessed at the predefined S1 level on fraction one, and all images were rigidly registered to the first fraction. Muscle area was averaged over three slices. Although this may introduce some uncertainty, consistent daily setup on the MR-linac and avoiding repeated S1 identification helped streamline the workflow and minimise manual input.

As PCa patients live longer, with over 90 % surviving 10 years post-treatment, it is increasingly important to assess quality of life outcomes such as fracture risk, fatigue, independence, and mobility [38]. Significant differences were observed between patients receiving 20# and 5# treatments, likely due to the longer treatment duration in the 20# group, which allowed more substantial changes. The additional stress of daily treatments over a month, compared to alternate-day treatments over two weeks, may have contributed. No significant differences in muscle or SAT metrics were observed between the 5# + ADT and 5# + noADT groups, though IMAT change was notable. As previously mentioned, IMAT is variable, and further work is needed to characterise it. This may reflect the potential influence of ADT’s negative impact on lean muscle mass, which has been reported in several studies [[14], [15], [16], [17], [18],^39^,^40^].

This study focused on changes within the MRgRT treatment window, a timeframe not previously explored. Most studies assess measurements at treatment initiation, after ADT completion (typically six months), or in follow-up imaging years later. One study reported muscle deterioration 14–20 weeks after ADT initiation, comparable to this study, where ADT began at least three months before RT [41]. Further research is needed to determine the timing of ADT-related muscle loss. In our cohort, 5# patients with and without ADT showed slight muscle gains, while 20# patients showed declines. Measurements were relative to the first RT fraction (∼10 days for 5#; ∼27 days for 20#), so muscle loss in ADT patients may have occurred before RT. While this method could apply to diagnostic, planning, or follow-up MRIs, we focused exclusively on on-treatment imaging. The absence of key baseline demographic and clinical data (e.g., comorbidities, lifestyle factors) limited our ability to adjust for confounders. Future work should account for these covariates to clarify additional drivers of BC changes.

As MRgRT becomes more widely available, automated BC analysis using on-treatment imaging offers valuable insights into treatment response. Two segmentation models achieved expert-level performance using on-treatment images of PCa patients. Significant differences in baseline values and treatment-related changes in muscle, SAT, and IMAT were observed across fractionation schemes and ADT status. These findings support non-invasive BC monitoring for early sarcopenia detection and timely adaptive interventions, with future work extending to multicentre cohorts to assess baseline variation and outcome associations.

## 6 Declaration of generative AI in scientific writing

During the preparation of this work the authors used ChatGPT 4.0 in order to enhance clarity and readability of the writing. After using this tool, the authors reviewed and edited content as needed and take full responsibility for the content of the publication.

## 7 Code availability

Scripts used in the development of the segmentation model can be found at: https://github.com/aaron-rankin/MRgRT-BodyComp.

## CRediT authorship contribution statement

**Aaron G. Rankin:** Conceptualization, Methodology, Software, Data curation, Formal analysis, Writing – original draft, Writing – review & editing, Visualization. **Dónal M. McSweeney:** Conceptualization, Methodology, Software, Supervision, Writing – review & editing. **Jim Zhong:** Data curation, Methodology. **Carlos Garcia Escobar:** Data curation, Methodology. **William Russell:** Data curation, Methodology. **Angela Davey:** Supervision, Writing – review & editing. **Marianne C. Aznar:** Funding acquisition, Supervision, Writing – review & editing. **Alan McWilliam:** Conceptualization, Data curation, Funding acquisition, Methodology, Supervision, Writing – review & editing.

## Declaration of competing interest

The authors declare that they have no known competing financial interests or personal relationships that could have appeared to influence the work reported in this paper.
