# Supplementary material for: Assessing body composition using auto-segmentations of muscle and subcutaneous adipose tissue in prostate cancer patients receiving magnetic resonance-guided radiotherapy
Source: Phys Imaging Radiat Oncol. 2025 Dec 6;36:100882. doi: 10.1016/j.phro.2025.100882 (PMC12753243; doi:10.1016/j.phro.2025.100882)
Supplement: Supplementary Data 1 [file mmc1.docx]

## **Supplementary Material**

1. **Image acquisition parameters**

*Supplementary Table 1 - Breakdown of parameters used to acquire the T2-weighted images during MR-guided radiotherapy.*

| Parameter | Value |
| --- | --- |
| Manufacturer | Philips |
| Model | Marlin |
| Sequence | T2 3D Tra 2min |
| Acquisition Type | 3D |
| Magnetic Field Strength (T) | 1.5 |
| In-plane Pixel Spacing (mm) | 0.83 x 0.83 |
| Rows | 480 |
| Columns | 480 |
| Slices | 300 |
| Spacing Between Slices (mm) | 1 |
| Slice Thickness (mm) | 2 |
| Repetition Time (ms) | 1536 |
| Echo Time (ms) | 276 |

1. **Model development**
   1. **Pipeline Schematic**

*
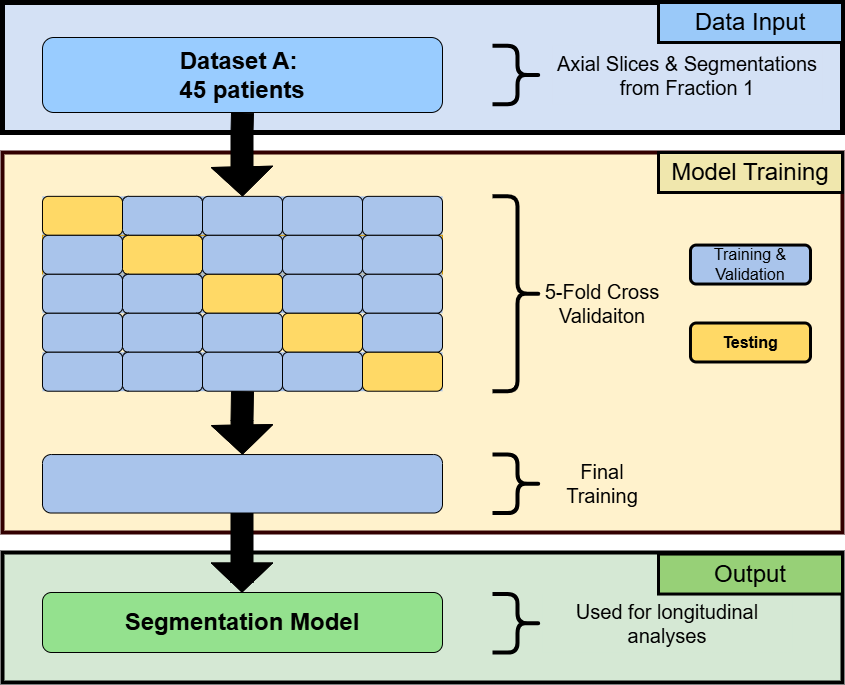
*

Supplementary Figure 1 – Pipeline diagram showing the steps taken to construct our segmentation model. An experienced radiologist segmented muscle and subcutaneous adipose tissue for a subset of 45 images – 15 patients from each treatment group and images were acquired at the first fraction of treatment. Cross-validation was used to train and validate the model. Model performance was assessed after each training fold. The final model was trained on all 45 images.

- 1. **Network Architecture & Model Training**

The segmentation model is based on an inhouse framework from McSweeney et al [1]. The network was built using the PyTorch and Segmentation Models PyTorch packages [2,3]. Our network architecture was based on the UNet framework with encoders from based on the Mixed Depthwise Convolutional Kernels (MixNet-M) first proposed by Tan and Le [4,5]. The architecture total is shown in Supplementary Table 2. We selected MixNet-M as the encoder backbone because it provides ImageNet pre-trained weights suitable for transfer learning to relatively small medical imaging datasets, while remaining computationally lightweight and effective at extracting both local and global image features.

**Model Inputs:** 2D axial slices (480 x 480) at the predefined S1 level with ROI labels (0: background, 1: muscle, 2: SAT, 3: BB [if applicable])

**Model Outputs:** Probability masks for each ROI stored as one-hot encoded masks after SoftMax.

**Training and Hyperparameters:**

- Optimizer: Adam
- Learning rate: 3 × 10⁻⁴
- Loss function: Combination of categorical cross-entropy and multiclass Dice loss
- Initialisation: ImageNet pre-trained weights for encoder
- Normalisation: All images were standardised to ImageNet mean and standard deviation [mean=(0.485,0.456,0.406), std=(0.229,0.224,0.225)]
- Data Augmentation: Random horizontal flips and rotations
- Batch size: 3
- Epochs: Maximum 200, with early stopping (Final model stopped after 114 epochs)
- Total parameters: 5,155,722
- Hardware: NVIDIA RTX 3090 GPU (24 GB VRAM)
- Model Inference time: 0.02 s per slice

**Network Architecture**

Supplementary Table 2 – Break down of network architecture used in segmentation model.

| - 1. Stage | Channels | | Resolution | | - 1. Components |
| --- | --- | --- | --- | --- | --- |
|  | Input Channels | Output Channels | Input | Output |  |
| Input | 3 | 24 | 480x480 | 240x240 | Conv (3x3, stride 2),  DW + PW |
| Encoder 1 | 24 | 16 | 240x240 | 120x120 | Inverted Residual Block x2 |
| Encoder 2 | 16 | 40 | 120x120 | 60x60 | Inverted Residual Block x4 |
| Encoder 3 | 40 | 80 | 60x60 | 30x30 | Inverted Residual Block x4 |
| Encoder 4 | 80 | 120 | 30x30 | 15x15 | Inverted Residual Block x4 |
| Encoder 5 | 120 | 200 | 15x15 | 8x8 | Inverted Residual Block x4 |
| Decoder 1 | 200 | 120 | 8x8 | 15x15 | Decoder Block + Skip Connection (Encoder 5) |
| Decoder 2 | 120 | 80 | 15x15 | 30x30 | Decoder Block + Skip Connection (Encoder 4) |
| Decoder 3 | 80 | 40 | 30x30 | 60x60 | Decoder Block + Skip Connection (Encoder 3) |
| Decoder 4 | 40 | 16 | 60x60 | 15x15 | Decoder Block + Skip Connection (Encoder 2) |
| Decoder 5 | 16 | 16 | 120x120 | 240x240 | Decoder Block + Skip Connection (Stem) |
| Head | 16 | 4 | 240x240 | 480x480 | Conv (1x1) |

Where in each Inverted Residual block is part of the “tu-mixnet-m” encoder from Segmentation Models package:

- - Expansion (1×1 Conv)
  - Depthwise Convolution (DW Conv)
  - Projection (1×1 Conv)
  - Residual Connection

And each Decoder Block is defined as:

- - Two Bilinear Upsample layers
  - Two 3×3 Convolutions
  - Skip Connection: Merges early spatial details with upsampled features.

1. **Body composition analysis pipeline**


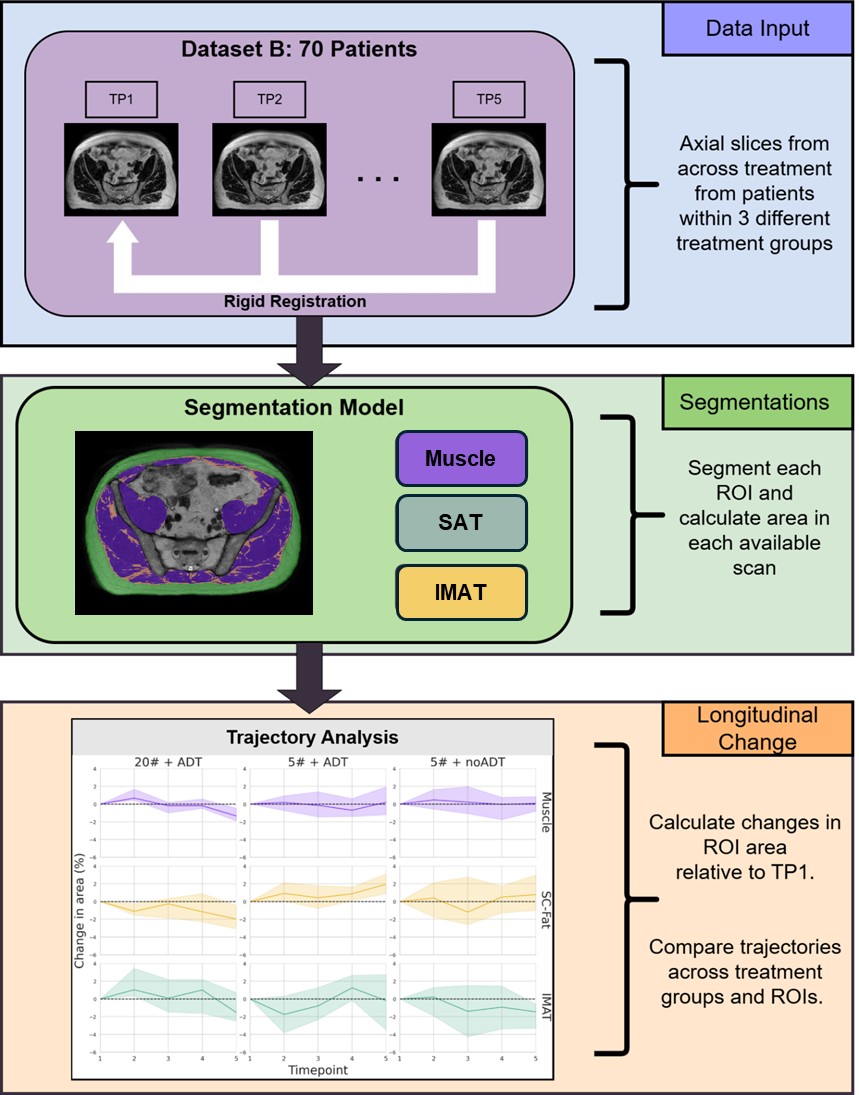


*Supplementary Figure 2 – Pipeline diagram showing steps involved in assessing changes in body composition across treatment. A total of 70 patients, each with 5 images across treatment were used for analysis. Images acquired after the first fraction of treatment were rigidly registered to the fraction 1 image to ensure segmentation of the same anatomical region. For each image, segmentations of muscle, subcutaneous adipose tissue (SAT) and intramuscular adipose tissue (IMAT) were performed at the S1 vertebral level and change in area, relative to the first fraction were computed.*

1. **
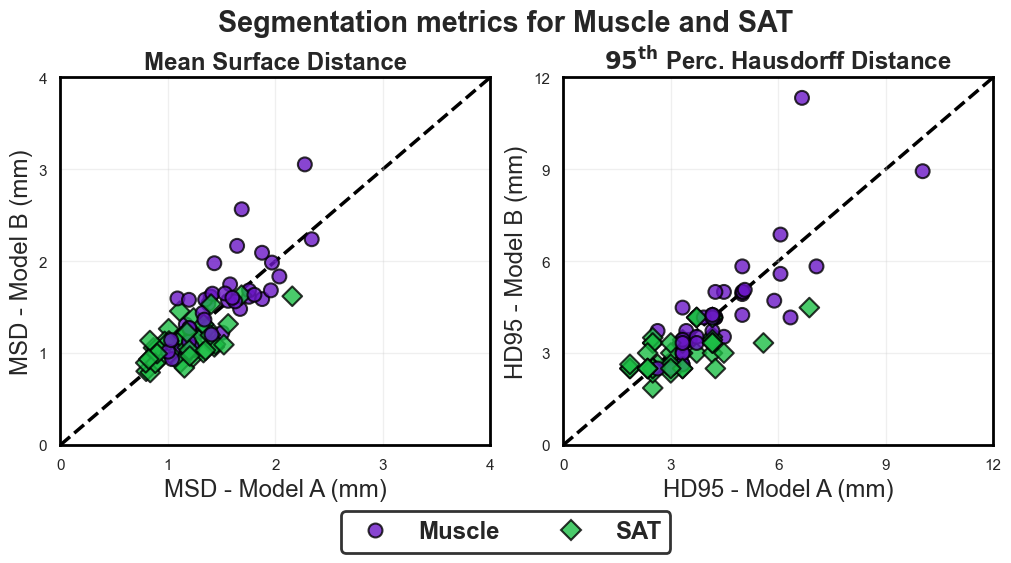
Model comparison with and without bowel and bone mask**

Supplementary Figure 3 - Identity plots for segmentation metrics evaluated during 5-fold cross validation investigating the impact of including the bowel and bone (BB) mask in model training. Model A was trained with the muscle and subcutaneous adipose tissue (SAT) and BB masks and Model B was trained with only muscle and SAT masks.

1. **Segmentation failure examples**


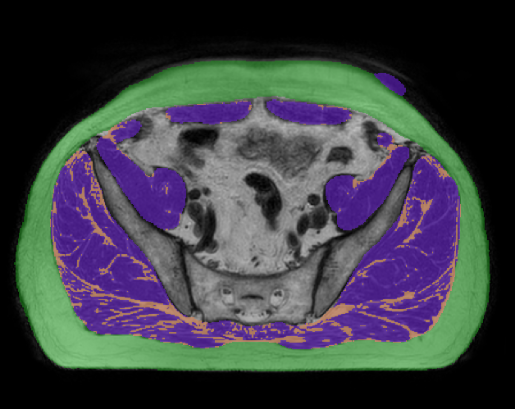
**
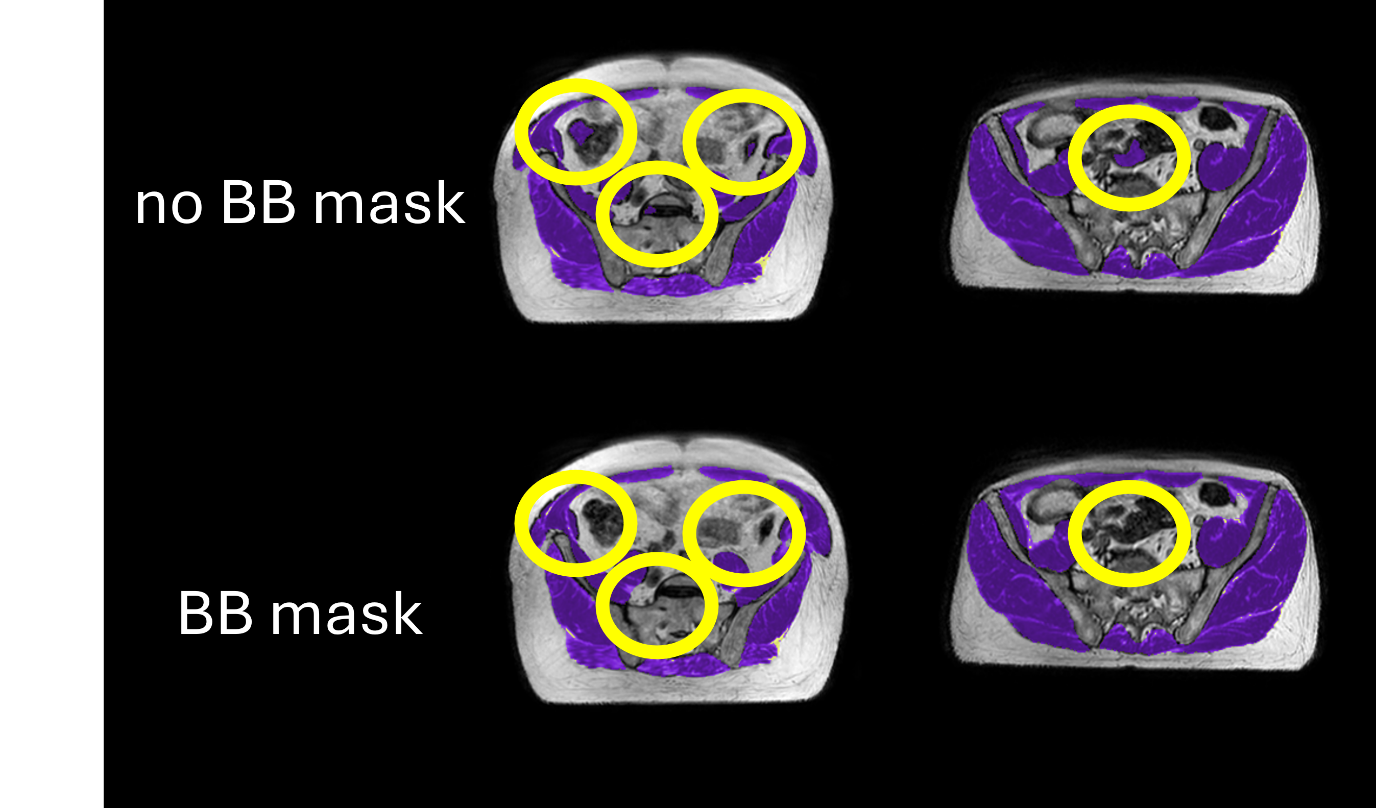
**

Supplementary Figure 5 – An example cases where the presence of edge artefacts with low intensity pixels were incorrectly segmented as muscle.

Supplementary Figure 4 – Two example cases where the inclusion of the BB mask improved segmentation of muscle. (top) Rings outline areas where, without the inclusion of the BB mask, bowel gas was incorrectly labelled as muscle. (bottom) Segmentations of the same scan using the model trained with the BB mask with rings used to highlight the corrected areas.

1. **
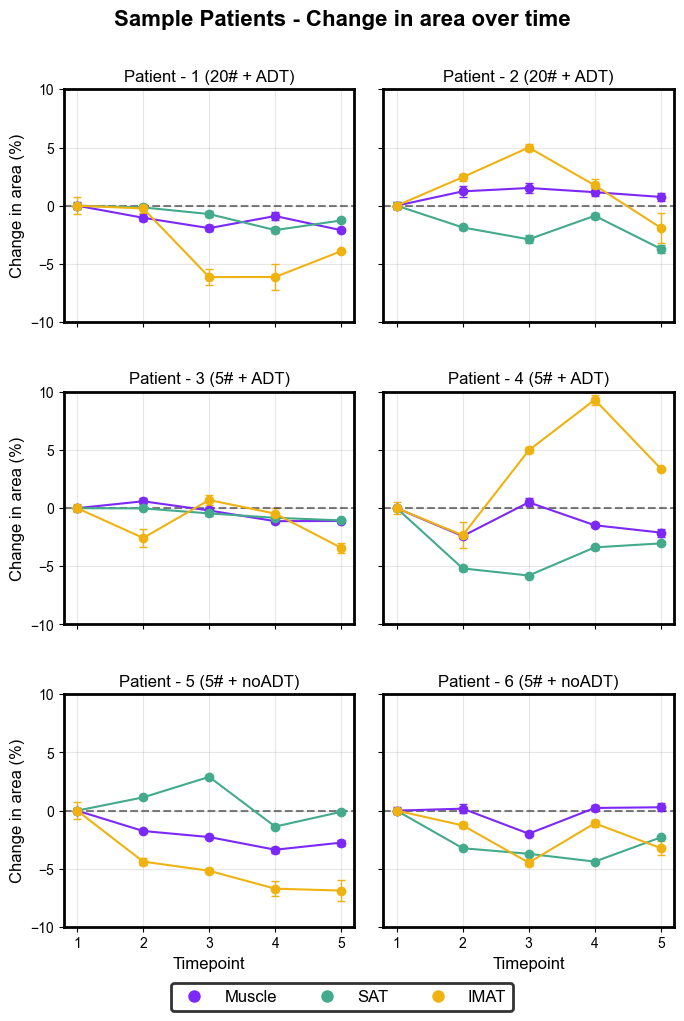
Example curves for ROI area changes during treatment**

Supplementary Figure 6 – ROI area trajectories across 2 patients from each treatment group. Values plotted are the mean ROI area change across S1 ± 1 slice determined at Timepoint 1. Images were collected weekly for the 20# + ADT group (median duration 27 days) and images were collected at each treatment fraction for both 5# groups (median duration 10 days).

1. **Region of interest areas at start and end of treatment**

| ROI | Group | Area at Start of Treatment (cm²)  [CI95] | Area at End of Treatment (cm²)  [CI95] | Change in Area (%)  [CI95] |
| --- | --- | --- | --- | --- |
|  | **20# + ADT** | 200 ± 6 | 198.21 ± 5.76 | -1.03 ± 0.44 |
|  |  | [189, 211] | [186.93, 209.49] | [-1.90, -0.17] |
| Muscle | **5# + ADT** | 195 ± 6 | 198.42 ± 6.03 | 0.56 ± 0.53 |
|  |  | [183, 207] | [186.59, 210.24] | [-0.48, 1.59] |
|  | **5# + noADT** | 202 ± 7 | 202.77 ± 7.26 | 0.18 ± 0.46 |
|  |  | [188, 217] | [188.54, 217.0] | [-0.72, 1.08] |
|  | **20# + ADT** | 228 ± 19 | 224.30 ± 19.09 | -1.87 ± 0.61 |
|  |  | [191, 265] | [186.9, 261.71] | [-3.08, -0.67] |
| SAT | **5# + ADT** | 192 ± 16 | 190.77 ± 16.05 | 0.63 ± 0.51 |
|  |  | [160.78, 223.25] | [159.3, 222.23] | [-0.36, 1.63] |
|  | **5# + noADT** | 170.43 ± 13.76 | 170.14 ± 14.27 | -0.62 ± 0.62 |
|  |  | [143.46, 197.39] | [142.18, 198.11] | [-1.82, 0.59] |
|  | **20# + ADT** | 34.65 ± 1.37 | 34.04 ± 1.38 | -1.78 ± 0.69 |
|  |  | [31.97, 37.33] | [31.34, 36.74] | [-3.12, -0.43] |
| IMAT | **5# + ADT** | 33.14 ± 1.23 | 33.98 ± 1.23 | 1.58 ± 1.11 |
|  |  | [30.72, 35.56] | [31.57, 36.38] | [-0.60, 3.76] |
|  | **5# + noADT** | 33.80 ± 1.32 | 33.23 ± 1.30 | -1.67 ± 1.00 |
|  |  | [31.22, 36.39] | [30.69, 35.77] | [-3.63, 0.30] |

**References**

[1] D. M. McSweeney *et al.*, ‘Transfer learning for data-efficient abdominal muscle segmentation with convolutional neural networks’, *Med. Phys.*, vol. 49, no. 5, 2022, doi: 10.1002/mp.15533.

[2] O. Ronneberger, P. Fischer, and T. Brox, “U-Net: Convolutional networks for biomedical image segmentation,” arXiv preprint arXiv:1505.04597, 2015.

[3] A. Paszke et al., ‘PyTorch: An Imperative Style, High-Performance Deep Learning Library’, Dec. 03, 2019, arXiv: arXiv:1912.01703. doi: 10.48550/arXiv.1912.01703.

[4] P. Iakubovskii, *qubvel-org/segmentation_models.pytorch*. (Mar. 23, 2025). Python. qubvel-org. Accessed: Mar. 23, 2025. [Online]. Available: https://github.com/qubvel-org/segmentation_models.pytorch

[5] M. Tan and Q. V. Le, “MixConv: Mixed depthwise convolutional kernels,” arXiv preprint arXiv:1907.09595, 2019.
